# Brain Volume Alterations and Cognitive Functions in Patients with Common Variable Immunodeficiency: Evaluating the Impact of Autoimmunity

**DOI:** 10.3390/jcm15020503

**Published:** 2026-01-08

**Authors:** Filiz Sadi Aykan, Duygu Akın Saygın, Fatih Çölkesen, Necdet Poyraz, Recep Evcen, Mehmet Kılınç, Eray Yıldız, Tuğba Önalan, Fatma Arzu Akkuş, Elif Erat Çelik, Cemile Buket Tuğan Yıldız, Ganime Dilek Emlik, Şevket Arslan

**Affiliations:** 1 Division of Clinical Immunology and Allergy, Department of Internal Medicine, Ankara Health Sciences University Gülhane Training and Research Hospital, 06010 Ankara, Türkiye; 2 Division of Anatomy, Department of Basic Medical Sciences, Faculty of Medicine, Necmettin Erbakan University, 42090 Konya, Türkiye; d.akin.42@hotmail.com; 3 Division of Clinical Immunology and Allergy, Department of Internal Medicine, Faculty of Medicine, Necmettin Erbakan University, 42090 Konya, Türkiye; drvefa42@hotmail.com (F.Ç.); tugbaonalan@gmail.com (T.Ö.); arzusmr@hotmail.com (F.A.A.); drelifcelik14@gmail.com (E.E.Ç.); arslansevket@hotmail.com (Ş.A.); 4 Department of Radiology, Faculty of Medicine, Necmettin Erbakan University, 42090 Konya, Türkiye; necdetpoyraz@gmail.com (N.P.); drdemlik@hotmail.com (G.D.E.); 5 Division of Clinical Immunology and Allergy, Department of Internal Medicine, Recep Tayyip Erdoğan University Training and Research Hospital, 53100 Rize, Türkiye; r_evcen@hotmail.com; 6 Division of Clinical Immunology and Allergy, Department of Internal Medicine, Batman Training and Research Hospital, 72060 Batman, Türkiye; drmehmetalerji@gmail.com; 7 Division of Clinical Immunology and Allergy, Department of Internal Medicine, Necip Fazıl City Hospital, 46100 Kahramanmaraş, Türkiye; drerayyldz@gmail.com; 8 Department of Neurology, Kahramanmaraş Sütçü İmam University, 46100 Kahramanmaraş, Türkiye; bukettugan@yahoo.com

**Keywords:** common variable immunodeficiency, brain volumes, cognitive functions, autoimmunity, neuroinflammation, cerebrum, cerebellum, basal ganglia

## Abstract

**Background:** Common variable immunodeficiency is a heterogeneous disorder characterized by defects in antibody production and immune dysregulation, associated with infections and autoimmunity. Although structural and cognitive effects of CVID on the central nervous system have attracted attention in recent years, studies jointly addressing volumetric brain imaging and neurocognitive evaluation remain limited. **Materials and Methods:** In this retrospective cross-sectional study, 35 patients with common variable immunodeficiency and 40 age- and sex-matched healthy controls were evaluated. Cognitive performance was assessed in all participants using the Montreal Cognitive Assessment. High-resolution T1-weighted brain magnetic resonance imaging scans underwent automated segmentation using the volBrain platform, yielding quantitative volumetric measurements of cortical, subcortical, and cerebellar structures, as well as ventricles and cerebrospinal fluid. Intergroup comparisons were performed using independent *t*-tests and analysis of variance. **Results:** MoCA scores were significantly lower in patients with CVID. Volumetric analysis revealed prominent reductions in the volumes of total brain tissue, gray matter, cerebrum, cerebellum, limbic system, thalamus, and basal ganglia. Paralleling these findings, cerebrospinal fluid and lateral ventricle volumes were increased. Additional volume losses were detected in CVID patients with low MoCA scores. In CVID patients with autoimmunity, volume loss affected broader areas. **Conclusions:** CVID appears to be associated with structural brain changes and cognitive impairments. Chronic inflammation and immune dysregulation may contribute to these neurodegenerative processes. Regular neurocognitive monitoring and further prospective studies are warranted in patients with CVID.

## 1. Introduction

Common variable immunodeficiency (CVID) is one of the most common primary immunodeficiencies (PIDs), characterized by hypogammaglobulinemia, impaired specific antibody responses, and variable T-lymphocyte dysfunction—a rare disorder of the immune system [1,2]. This condition not only predisposes patients to recurrent infections but is also associated with various systemic complications, including autoimmune diseases, inflammatory disorders, and malignancies [3,4,5].

Magnetic resonance imaging (MRI) is a preferred non-invasive modality for the diagnosis and monitoring of neurological pathologies. Advanced tools such as the volBrain software (https://volbrain.net/, Polytechnic University of Valencia, Valencia, Spain), which automatically quantifies brain structure volumes, play a pivotal role in the effective volumetric analysis of neuroimaging data [6,7,8]. VolBrain is a free online platform that delivers automatic, objective, and accurate brain volume measurements from researchers’ MRI scans without requiring local infrastructure [7,8]. Detection of alterations in brain volumes, including subcortical structures, can provide valuable insights for the early diagnosis, prevention, and management of CVID-associated neuropsychiatric disorders [6].

While the effects of CVID on the immune system predominate, recent studies suggest that the disease may also exert potential impacts on the central nervous system (CNS), particularly the brain [4,9,10]. The presence of concomitant autoimmune diseases is thought to contribute to this clinical picture [4,11]. However, the number of studies examining structural and cognitive brain abnormalities in CVID patients remains quite limited. This scarcity highlights the need for advanced research to more clearly define the cognitive and neurological consequences of immune dysfunction [10,12]. Elucidating this relationship is critically important for the comprehensive management of CVID and improving patients’ quality of life.

The objective of this study is to assess cognitive functions in patients diagnosed with CVID using the Montreal Cognitive Assessment (MoCA), to evaluate structural and organic brain pathologies via MRI, and to quantify brain volumes with the volBrain software. Furthermore, through comparisons with healthy control (HC) group, it aims to comprehensively investigate the effects of CVID on brain structure, volumetrics, and cognitive functions, as well as the role of autoimmunity.

## 2. Materials and Methods

### 2.1. Study Description

This study was designed as a retrospective cross-sectional study, and the minimum required sample size was determined using the a priori sample size calculation module of G*Power version 3.1.9.7. It was calculated that a statistical power of 0.95 could be achieved with a medium effect size of 0.5, a 95% confidence interval, and a significance level (α) of 0.05. These parameters were selected to ensure robust statistical power and reliability of the results [13,14].

### 2.2. Study Design

This study was designed as a retrospective cross-sectional investigation reviewing the medical records and archived brain MRIs of 85 patients diagnosed with CVID who were followed at the Adult Immunology and Allergy Clinic of Necmettin Erbakan University Faculty of Medicine between January 2012 and November 2023. The aim was to comprehensively analyze clinical, cognitive, and imaging data to evaluate structural brain changes in patients with CVID.

### 2.3. Participants

The patient group comprised 35 individuals (18 men, 17 women) aged ≥18 years, diagnosed with CVID according to the diagnostic criteria of the European Society for Immunodeficiencies [15], with no history of neurological disorders and possessing T1-weighted brain MRIs at 1 × 1 × 1 mm^3^ resolution. The control group consisted of 40 healthy individuals (20 men, 20 women) with no diagnoses from the neurology outpatient clinic, no CVID or other comorbidities, and T1-weighted MRIs at the same resolution.

### 2.4. Inclusion and Exclusion Criteria

Only individuals aged 18 years or older, with complete brain MRI scans and who had completed the MoCA, were included in the study. Individuals younger than 18 years, those without available brain MRI data, patients diagnosed with neurological disorders (all comorbidities for the HC group), and those unable to complete cognitive evaluation via the MoCA test were excluded. Additionally, in the CVID group, potential confounding medical conditions and treatments that could impact cognitive performance and brain volumetry were meticulously evaluated through a detailed review of all participants’ medical records.

CVID patients with thyroid disorders, uncontrolled endocrine-metabolic diseases (including diabetes mellitus), chronic renal or hepatic failure, clinically significant pulmonary diseases leading to respiratory insufficiency, and diagnosed sleep disorders requiring device usage were excluded from the study. Furthermore, patients receiving long-term systemic corticosteroid therapy at the time of MRI acquisition, as well as those using antipsychotic, antidepressant, anxiolytic, or other psychotropic medications, were also excluded.

Data regarding alcohol consumption, recreational substance use, and excessive caffeine intake were obtained from routine outpatient follow-up records and verified by the patients themselves. No documented substance use or chronic alcohol consumption that could significantly impact cognitive assessments and MRI volumetric measurements was identified in any of the study participants.

### 2.5. Clinical and Cognitive Assessment

Demographic data for all participants, including age, sex, education level, height, weight, body mass index (BMI), and dominant hand, were recorded using standard forms developed for this study. For the CVID group specifically, disease duration, presence of autoimmunity, comorbidities, treatments received, and immunological parameters at the time of diagnosis (immunoglobulin levels and lymphocyte subsets) were also assessed. Cognitive functions were evaluated in both groups using the MoCA. In the CVID group, the effects of serum IgG levels, the interval from the onset of first symptoms to the initiation of immunoglobulin replacement therapy (IgRT), and the duration of IgRT on cognitive performance and brain volumes were additionally examined via correlation analyses.

### 2.6. Imaging and Volumetric Analysis

All brain MRIs were retrieved from the hospital’s Picture Archiving and Communication System (PACS). A cross-sectional design was employed to investigate structural brain alterations. Brain parcellation was performed using volBrain on diffusion tensor imaging data to obtain the volumes of individual brain structures [7]. For all participants, the cerebrum, cerebellum, thalamus, limbic system structures, basal ganglia, lateral ventricles, and cerebrospinal fluid spaces (CSF) were analyzed in detail.

The basal ganglia structures were evaluated individually due to their well-defined anatomical boundaries, high segmentation reliability, and sensitivity to inflammatory processes as subcortical nuclei. In contrast, the cerebral cortex—characterized by high variability, numerous subregions, and an elevated risk of false positives from over-parcellation in small samples—was analyzed globally in terms of total cortical volume. This deliberate methodological choice was made to preserve statistical power and yield clinically meaningful, reliable results [16,17].

### 2.7. Multidisciplinary Evaluation

The CVID diagnoses, MoCA results, MRI scans, and volumetric analyses of the images using volBrain were jointly evaluated by a multidisciplinary team comprising experienced researchers in immunology, radiology, anatomy, and neurology. This holistic approach facilitated an integrated interpretation of both cognitive performance and structural brain changes.

### 2.8. Assessment Tools

#### 2.8.1. Montreal Cognitive Assessment (MoCA)

The scale, developed by Nasreddine and colleagues [18], assesses various cognitive domains, including attention and concentration, executive functions, memory, language, visuospatial skills, abstraction, and orientation. The MoCA is a screening tool specifically designed to detect early stages of cognitive impairment. Its Turkish validation and reliability study was conducted by Selekler, Cangöz, and Uluç. The scale yields a minimum score of 0 and a maximum score of 30, with a cutoff point of 21; scores of 21 or higher are considered within normal limits [19].

#### 2.8.2. Brain MRI Protocol

The MRI protocol (3 Tesla) included high-resolution 3D magnetization-prepared rapid gradient-echo (MPRAGE) T1-weighted sequences for anatomical imaging. These sequences comprised retrospective data acquired during routine clinical procedures. Imaging parameters were as follows: sagittal orientation, repetition time (TR) of 1900 ms (2.84 s), flip angle of 15°, echo time (TE) of 2.67 ms, field of view (FOV) of 256 mm^2^, matrix size of 256 × 256, 160 slices each 1 mm thick, and isotropic spatial resolution of 1 × 1 × 1 mm^3^ [20].

#### 2.8.3. VolBrain Method and Analysis

In this study, volBrain (https://volbrain.net/), an open-access platform, was utilized for the automatic segmentation of various brain structures. Total brain volumetric analysis was performed across study groups using default volBrain T1-weighted volumetric images. To facilitate volume calculations, MRI scans were converted to ‘gz’ or ‘rar’ formats. The processing steps were as follows: The procedure began by opening a ‘DICOMDIR’ file using RadiAnt DICOM viewer software (Version 2023.2; Medixant, Poznań, Poland). Subsequently, high-resolution T1-weighted 3D MPRAGE images were accessed via MRIcron software (Version 1.0; Chris Rorden, University of South Carolina, Columbia, SC, USA) for anatomical visualization, generating a compressed ‘gz’ file in FSL format. These ‘gz’ files were then uploaded to the volBrain web interface, where registration was completed, and the files were submitted for processing. Upon completion of the upload, the system initiated volumetric analyses for all brain regions. The resulting volumetric data were delivered to the registered email address in Portable Document Format (PDF) and Comma-Separated Values (CSV) files [20,21].

**Figure 1:**
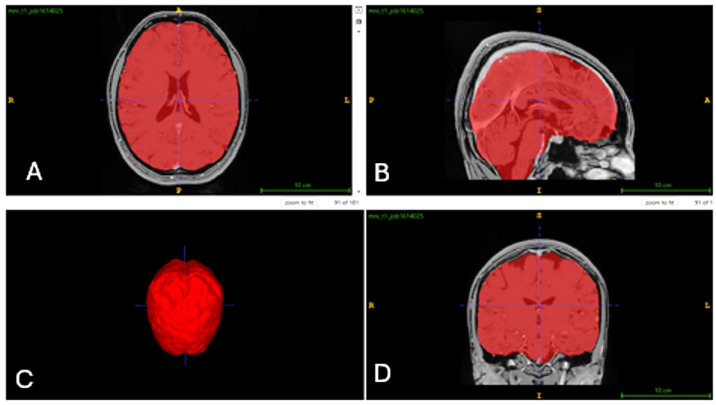
Brain volumetric analysis and 3D reconstruction on MRI scans. ((**A**): axial, (**B**): sagittal, (**C**): 3D image, (**D**): coronal).

**Figure 2:**
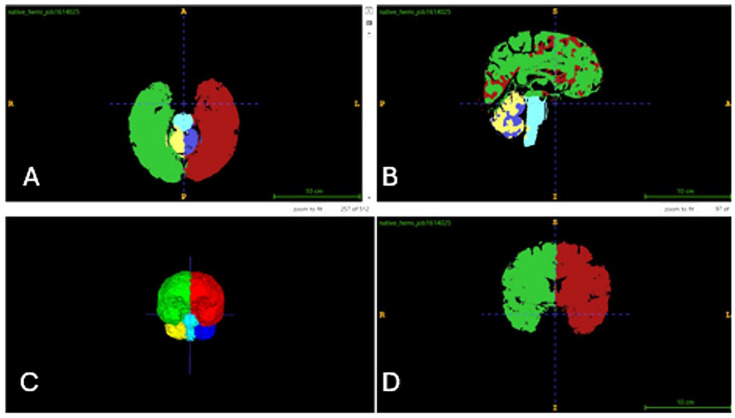
Automatic segmentation of brain structures at the hemispheric and lobular levels ((**A**): axial, (**B**): sagittal, (**C**): 3D image, (**D**): coronal).

**Figure 3:**
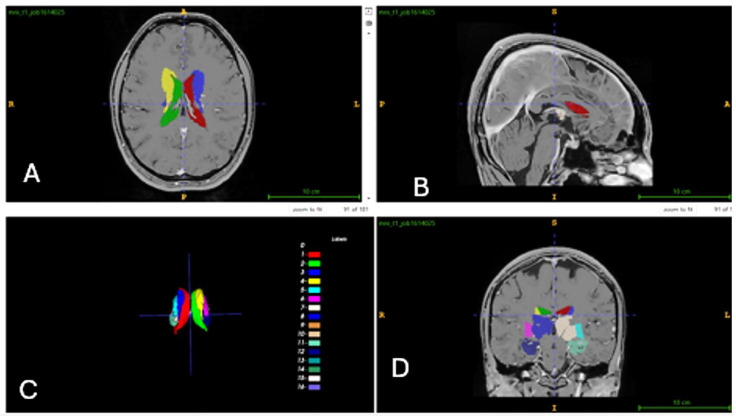
Thalamic nuclei segmentation (**A**): axial, (**B**): sagittal, (**C**): 3D image, (**D**): coronal.

In this study, the AssemblyNet module of volBrain was utilized. AssemblyNet is a large convolutional neural network (CNN) ensemble designed for the segmentation of 3D whole-brain MRIs. Volumetric values of all brain regions were measured in cm^3^ and as percentages, with total right-to-left ratios calculated. A total of 512 distinct data points were obtained from each participant. Measurements encompassed the brain, intracranial (IC) volume, white matter (WM) and gray matter (GM) in the cerebrum and cerebellum, limbic system structures, CSF and lateral ventricles, thalamic nuclei, and “deep thalamus” regions within the basal ganglia [21]. The volume analyses and segmentations for the control group are presented in Figure 1, Figure 2 and Figure 3.

### 2.9. Statistical Analysis

All statistical analyses were performed using IBM SPSS Statistics for Windows, version 21.0 (IBM Corp., Chicago, IL, USA). The normality of continuous variables was evaluated by examining skewness and kurtosis coefficients, as well as by the Shapiro–Wilk test. Variables with skewness and kurtosis values between −1 and +1 were considered to be normally distributed [22].

All morphometric measurements were obtained three times for each participant by the same investigator at different time points. For statistical analyses, the mean of the three measurements was used. The reliability of repeated measurements was assessed using repeated-measures analysis of variance (ANOVA). Bonferroni correction was applied to control for Type I error. No statistically significant differences were observed among repeated measurements (*p* > 0.05), confirming measurement consistency.

Descriptive statistics were expressed as mean ± standard deviation (SD) for continuous variables and as number (*n*) and percentage (%) for categorical variables. Minimum (min.) and maximum (max.) values were also reported where appropriate. Comparisons of morphometric measurements between genders were performed using the independent-samples *t*-test for normally distributed variables. Right–left side comparisons were conducted using the paired-samples *t*-test. Comparisons among the HC group and CVID subgroups with and without autoimmunity were performed using one-way analysis of variance (ANOVA) followed by Tukey’s post hoc test.

Correlations between morphometric measurements and demographic variables, including age, handedness, and BMI, were evaluated using Pearson correlation analysis. The strength of correlation coefficients (r) was interpreted according to Cohen’s criteria as weak (0.10–0.29), moderate (0.30–0.49), and strong (≥0.50) [23].

Associations between clinical and immunological variables (diagnostic delay, IgRT duration, and serum IgG levels) and MRI-derived brain volumetric parameters were assessed using Pearson correlation analysis for normally distributed variables. Two-tailed *p* values < 0.05 were considered statistically significant.

## 3. Results

In our study, two groups comprising 35 patients with CVID and 40 HC, totaling 75 individuals, were evaluated. The mean age of the CVID group was 38.46 years, whereas that of the HC group was 33.9 years. Both groups demonstrated comparable BMI, hand dominance, and education levels. MoCA scores evaluating cognitive functions were significantly lower in the patient group (*p* = 0.005). All CVID patients were receiving IgRT, and autoimmune conditions were present in 51.4% of them (Table 1).

Volumetric (cm^3^) and proportional (%) values of brain structures were calculated from the brain MRIs of the CVID (*n* = 35) patients and HC (*n* = 40) group. A significant reduction in total brain tissue volume was detected in the CVID group, which was statistically significant (*p* = 0.001). Total GM volume was measured as 717.51 cm^3^ (%50.76) in the HC group, and 685.3 cm^3^ (%48.45) in the CVID group. This finding indicates an approximately 2% reduction in the CVID group, with the difference being statistically significant (*p* = 0.002). Furthermore, similar significant reductions were observed in both total volumes and GM volumes of the cerebrum and cerebellum. Significant reductions were also noted in the total volumes of limbic system structures (hippocampus, amygdala), thalamus, and basal ganglia (caudate, putamen, globus pallidus, accumbens) in the CVID group (Table 2).

In contrast, marked increases were observed in CSF and lateral ventricle volumes in the CVID group. CSF volume was 157.88 ± 53.53 cm^3^ (%11, 13 ± 3.4) in the HC group and 199.25 ± 59.9 cm^3^ (%14, 11 ± 4.08) in the CVID group. Total lateral ventricle volume was 13.69 ± 8.88 cm^3^ (% 0.97 ± 0.65) in the CVID group and 9.01 ± 4.87 cm^3^ (%0.64 ± 0.34) in the HC group. These increases in the CVID group were statistically significant (Table 2).

In conclusion, no significant differences were detected between the two groups in IC space volume, WM, or brainstem volume, whereas increases were observed in CSF and lateral ventricle volumes and significant reductions in the volumes of other brain regions (Table 2).

The volumes of right- and left-hemispheric structures in the CVID and HC group were evaluated using intra-group comparisons. In the CVID group, significant reductions were observed in both hemispheres compared to the HC group in total cerebellar volume, cerebellar GM and WM volumes, and the volumes of the caudate nucleus and globus pallidus basal ganglia, alongside a significant increase in lateral ventricular volume. Other differences were similar between the two groups (Table 3).

In the CVID group, no statistically significant correlations were observed between diagnostic delay and any of the brain volumetric parameters (all *p* > 0.05). The duration of IgRT demonstrated a significant positive correlation with CSF volume (r = 0.341, *p* = 0.041). Serum IgG levels showed a significant positive correlation with cerebellar volume (r = 0.351, *p* = 0.036). No significant correlations were found between serum IgG levels and other global or regional brain volumetric parameters (Table 4).

In the CVID group, brain tissue volumes were compared between individuals with normal and low cognitive function, as defined by MoCA scores. In the low MoCA subgroup, IC space volume, total WM volume, cerebrum WM volume, and cerebellum GM volume were significantly lower (*p* = 0.028, 0.014, 0.014 and 0.048, respectively) (Table 5).

CVID patients were stratified according to the presence of concomitant autoimmune conditions, with no significant differences observed in age, sex, BMI, education level, or MoCA scores between subgroups. Immunoglobulin levels and lymphocyte subsets were comparable (Table 6).

When brain region volumes of CVID subgroups with and without autoimmunity were separately compared to the HC group, significant differences were observed. Notably, more brain regions were affected in the CVID subgroup with autoimmunity. Compared to the HC group, the CVID subgroup with autoimmunity exhibited significant reductions in total cerebrum and cerebellum volumes, alongside significant increases in CSF and lateral ventricle volumes. In the CVID subgroup without autoimmunity, significant reductions in total GM and cerebrum GM volumes were observed compared to the HC group. For other volume changes, either no differences were detected, or similar differences were found in both CVID subgroups relative to the HC group (Table 7).

## 4. Discussion

This study aimed to evaluate brain volumes and cognitive functions in patients with CVID by comparing them to HC group and to investigate the impact of concomitant autoimmunity. Our findings demonstrate volumetric reductions in numerous brain structures among CVID patients, significantly lower MoCA scores, and adverse effects of concomitant autoimmune conditions on both cognitive functions and brain volumes. These results support the concept that CVID is a systemic disease that also affects the CNS.

CVID is the most common symptomatic PID, with an estimated incidence of 1 in 50,000 to 20,000 [24]. Recurrent sinopulmonary infections affect 70–80% of patients with CVID and represent the most characteristic primary manifestation [25]. Autoimmune disorders occur in approximately 30% of CVID cases and can occasionally manifest as the initial presentation of the disease in the absence of infection [2,26]. Due to the heterogeneous nature of the condition, multiple organ systems may be involved; however, its effects on the neurological system—which substantially influences quality of life—remain inadequately characterized [10].

Neurological complications in patients with CVID are highly diverse and may arise from various pathophysiological mechanisms, including infection, inflammation, autoimmunity, and even malignancy [4]. Furthermore, the impact of CVID on cognitive functions is increasingly recognized. Studies have demonstrated that adult CVID patients obtain significantly lower scores on cognitive function questionnaires compared to the general population, indicating cognitive impairment in these patients [10]. Although the precise etiologies remain incompletely understood, CVID—being closely associated with autoimmunity—is postulated to trigger inflammatory responses that directly affect the nervous system [12]. This notion is supported by prior case series reporting that concomitant autoimmune diseases contribute to this clinical picture [4,11]. In the present study, despite comparable demographic characteristics (age, BMI, hand dominance, education level) between the CVID and HC group, MoCA scores were markedly lower in the CVID group. These findings align with existing evidence from the literature that CVID extends beyond an immunological disorder and may impair cognitive processes. Moreover, the prevalence of CVID-autoimmune disease (51.4%) comorbidity in this cohort exceeded that reported in the literature, thereby supporting the hypothesis that concomitant autoimmune processes may precipitate cognitive decline. Specific evidence regarding the direct impact of IgRT on cognitive functions in CVID patients is lacking in the literature. Given that all patients received IgRT owing to the nature of the disease, no inferences could be drawn concerning its influence on current cognitive status. Nevertheless, the results of this study are anticipated to delineate novel research avenues in this domain.

The brain is embryologically and anatomically divided into three primary regions: the forebrain (prosencephalon), midbrain (mesencephalon), and hindbrain (rhombencephalon) [27]. In the present study, the volumes of the forebrain and hindbrain regions—which are more closely associated with cognitive functions—were comprehensively evaluated, and their relationships with cognitive functions and autoimmunity were investigated. Additionally, total IC space and brain tissue volumes, as well as total CSF volumes, were measured. Due to the complex structure of the brain, the results are discussed below under separate headings:

### 4.1. Evaluation of Intracranial Space, Total Brain Tissue Volume, and Gray Matter–White Matter Changes

The involvement of the brain in CVID has been increasingly recognized in recent years. Prior studies and case reports have demonstrated reductions in brain tissue volume and GM volume [4,10,12]. The underlying causes of CNS impairment are thought to include, beyond recurrent infections characteristic of CVID, autoimmunity and inflammatory disorders [4,5]. Systemic inflammation has been shown to induce neuroinflammation and neurodegeneration resulting from neuronal damage, as cytokines cross the blood–brain barrier or directly reach the brain parenchyma. This suggests that systemic inflammation in CVID causes cortical atrophy in the brain and supports the neuroinflammatory nature of the disease [28]. Furthermore, previous studies on conditions such as multiple sclerosis and Alzheimer’s disease, which are characterized by GM reduction, indicate that CVID may also involve early neurodegenerative processes [29,30]. Consistent with the literature, the present study demonstrated significant reductions in total brain tissue volume and, particularly, the GM volume ratio in the CVID group. The volume losses identified in our study represent the structural reflection of systemic immune dysregulation and neuroinflammatory processes on the CNS in CVID, thereby supporting neurodegenerative mechanisms.

### 4.2. Evaluation of Forebrain Volume Changes

#### 4.2.1. Evaluation of Cerebrum Volume Changes

In chronic inflammatory conditions such as CVID, elevated systemic inflammatory mediators trigger neuroinflammation [31,32]. Neuroinflammation can disrupt neuronal signaling through microglial activation and even induce neuronal apoptosis, leading to reductions in GM volume [32]. Recurrent infections and autoimmunity—both common in CVID patients—can initiate these inflammatory responses that directly impact the nervous system [4,9,11]. Furthermore, autoimmune processes may contribute to neurological complications and brain volume alterations by promoting the formation of autoantibodies against neurons and glial cells or through T-cell-mediated attacks [11]. In this study, significant reductions were observed in both the total volume and GM volume of the cerebrum in the CVID group. These volume losses are consistent with prior studies and bolster the hypothesis that they arise from neuroinflammation and neurodegeneration associated with concomitant autoimmune conditions and recurrent infections.

#### 4.2.2. Evaluation of Limbic System, Thalamus, and Basal Ganglia Volume Changes:

These brain regions play critical roles in memory, mood, motor control, and other cognitive functions [11]. Hippocampal volume loss is commonly observed, particularly in the early stages of cognitive impairment, and plays a central role in neurodegenerative conditions such as Alzheimer’s disease [33,34]. Previous studies have directly associated reductions in hippocampal volume with the cognitive dysfunction and memory impairments observed in CVID patients [4,10]. Volume reductions in the amygdala have been suggested to be related to mood disorders or anxiety symptoms that may occur in CVID patients [35,36]. A study highlighting the importance of the thalamus in neuropsychiatric functions demonstrated that thalamic volume alterations in schizophrenia patients are associated with symptom severity and brain volume abnormalities [37]. Other studies evaluating the basal ganglia (caudate, putamen, globus pallidus, nucleus accumbens) have reported that volume reductions in these structures may be related to motor coordination deficits, bradykinesia, or decision-making difficulties observed in CVID patients. In the present study, consistent with the literature, significant reductions were identified in the total volumes of limbic system structures, the thalamus, and the basal ganglia [4,11]. These volume losses are postulated to be associated with the cognitive dysfunction and neuropsychiatric manifestations reported in CVID patients [10]. Our findings reinforce the notion that CVID is a complex systemic disorder that also impacts neurological and cognitive processes.

### 4.3. Evaluation of Hindbrain Volume Changes

Previous studies have demonstrated cerebellar atrophy in CVID patients, with chronic inflammation and immune dysregulation implicated as potential underlying mechanisms [31,32]. The cerebellum is known to be particularly vulnerable to immune-mediated damage in PID disorders, and cerebellar atrophy has been reported as the most common structural manifestation of CVID [38]. In the present study, significant reductions in both total and GM volumes of the cerebellum were observed in the CVID group, indicating that the neurodegenerative effects of the disease extend to critical centers for cognition and motor control, such as the cerebellum. Additionally, the positive correlation observed between serum IgG levels and cerebellar volume in our study supports the potential neuroprotective role of humoral immunity [39]. Furthermore, the lower cerebellar GM volume in patients with low MoCA scores supports other studies reporting the adverse effects of these structural changes on cognitive functions [4,10,40].

### 4.4. Lateral Ventricles and Cerebrospinal Fluid Volume Changes

In conditions such as aging or neurodegenerative diseases, decreases in brain volume lead to compensatory expansion of the ventricles and subarachnoid space, resulting in increased CSF volume [41,42]. In our study, the positive correlation between IgRT duration and CSF volume may reflect progressive subclinical parenchymal volume loss accompanying a longer disease course. Treatment duration may also represent a surrogate marker of disease chronicity, older age, or more severe phenotypes, and therefore, this finding should be interpreted cautiously.

Reductions in brain tissue volume, particularly GM, indicate neuronal and glial cell loss, whereas increases in CSF and ventricular volumes serve as indirect indicators of this tissue loss. A study in patients with PID reported CNS findings such as cerebral atrophy and hydrocephalus [38]. Similarly, in Crohn’s disease, another chronic inflammatory condition, systemic inflammation has been associated with reductions in GM and WM volumes, accompanied by changes related to CSF volume [43]. These observations suggest that chronic inflammation in CVID may influence brain morphology through analogous mechanisms. Furthermore, in models of chronic hydrocephalus, ventricular dilatation has been positively correlated with the severity of neuroinflammation. Consistent with the literature, the present study identified increases in CSF and ventricular volumes [44]. Our results support the existence and functionality of neuroinflammation and compensatory mechanisms. Additionally, ventricular enlargement and increased CSF volume may underlie one of the pathological mechanisms contributing to cognitive dysfunction in CVID patients [4,10].

### 4.5. Specific Evaluation of Right and Left Cerebral Hemisphere Volumes

In this study, unlike the control group, significant reductions were identified in the right and left cerebral hemispheres, in the total volume as well as GM and WM volumes of the cerebellum, and in the volumes of the caudate nucleus and globus pallidus of the basal ganglia in the CVID group, whereas a significant increase was observed in the lateral ventricle volume. These findings demonstrate the effects of CVID on the CNS at the level of specific brain regions, in addition to the disease’s general neurodegenerative impacts. Volume losses in specific regions of the cerebellum and basal ganglia provide a concrete neuroanatomical basis for motor and cognitive dysfunctions [4,10,38]. These results highlight the importance of neurological monitoring and neurocognitive assessment in CVID management.

### 4.6. Relationship Between Cognitive Functions and Brain Volumes

Brain volumetric changes can precede clinical cognitive symptoms. In particular, numerous studies have shown that structural alterations in the brain begin years before the onset of clinical symptoms in neurodegenerative diseases [45,46]. Neurological symptoms in patients with CVID are known to impose a significant burden and adversely affect quality of life. A previous study using the Neuro-QoL cognitive function questionnaire demonstrated that cognitive function is impaired in CVID patients compared with the general population [10]. Other studies have also reported mild cognitive impairments in CVID [4,10]. Consistent with prior research, MoCA scores were significantly lower in the CVID group in this study. In patients with low scores, significant reductions were observed in cerebrum WM and cerebellum GM volumes. These findings underscore the importance of WM in cognitive network connectivity [47]. Therefore, this study highlights the value of early neurocognitive screening in CVID patients.

### 4.7. Relationship Between Autoimmunity and Brain Volume

Previous studies have reported that systemic inflammation may affect the CNS and trigger neurodegenerative changes [48,49,50]. The observation of more pronounced volume losses in CVID patients with autoimmunity represents one of the most important findings of this study. This finding strengthens the hypothesis that autoimmune processes may contribute to neurodegeneration as an additional mechanism. This phenomenon can be explained by more aggressive immune dysregulation, the cerebral effects of systemic inflammation, and neuroinflammatory processes associated with autoimmunity [51]. In this context, the present study may provide one of the few comprehensive datasets demonstrating that the autoimmune subphenotype inflicts more pronounced damage on the brain.

### 4.8. Strengths and Limitations

This study has several limitations. First, the cross-sectional design conducted at a single center with a relatively small sample size may limit the generalizability of the findings. Second, potential temporal discrepancies between MRI acquisitions and cognitive testing necessitate cautious interpretation of the relationships between these datasets. Third, although major medical and pharmacological confounding factors were screened and excluded, the retrospective design of the study precluded systematic standardization of certain potential lifestyle and clinical variables, such as lipid-lowering medication use, mild asthma, daily caffeine consumption, and minor variations in sleep quality. Consequently, residual confounding effects of unmeasured lifestyle factors cannot be entirely ruled out. Finally, while cerebral cortex volume was evaluated globally, detailed parcellation of cortical lobes was not performed. Although the lack of lobe-based assessment of cortical structures can be considered a limitation, this was a deliberate methodological choice to reduce false positives in small sample sizes and enhance the reliability of the results.

However, despite these limitations, the study also possesses several notable strengths. The utilization of high-resolution volumetric MRI techniques, the implementation of advanced automated segmentation via volBrain software, the integrated analysis of cognitive and structural data, and the comparative evaluation against HC group have substantially enhanced the scientific value and quality of the study.

In this context, future prospective studies with larger sample sizes, in which all potential confounding factors are rigorously controlled through standardized exclusion criteria, are anticipated to make substantial contributions to more clearly elucidating the structural brain correlates of cognitive impairment in CVID. Furthermore, employing advanced cortical parcellation techniques to perform detailed volumetric analyses at the lobar and sublobar levels will provide significant insights into the regional neuroanatomical correlates of cognitive dysfunction in CVID.

## 5. Conclusions

In our study, significant reductions were observed, particularly in GM, limbic system structures, thalamus, basal ganglia, and cerebellum volumes in the CVID patient group. Paralleling these findings, increases in CSF and lateral ventricle volumes were noted, supporting the observed volume losses. Furthermore, this study robustly demonstrates that cognitive impairment is a significant clinical issue in CVID patients and can be objectively assessed using the MoCA test. Despite the limited literature, our findings corroborate that neurological effects in CVID often arise from neurodegenerative changes triggered by chronic inflammation secondary to infections, autoimmune inflammation, and immune dysregulation. In light of these results, regular neurocognitive evaluations should be implemented in CVID patients, with those exhibiting autoimmunity monitored as a high-risk subgroup. Future research investigating neuroinflammation-targeted treatment strategies, along with prospective, longitudinal studies enriched with neuropsychiatric assessments, is critically important for better understanding and management of this complex disorder.

## Figures and Tables

**Table 1:** Comparison of Demographic and Clinical Characteristics between CVID and HC Groups.

	CVID Group (*n* = 35)	HC Group (*n* = 40)	*p* Value
Age (Mean ± SD)	38.46 ± 11.24	33.9 ± 9.41	0.063
Gender, *n* (%) Female Male	17 (48.57) 18 (51.42)	20 (50) 20 (50)	0.902
BMI (Mean ± SD)	24.97 ± 4.3	24.17 ± 3.63	0.394
BMI assessment, *n* (%) Normal Overweight Grade 1 Obese	21 (60) 8 (22.9) 6 (17.1)	26 (65) 6 (15) 8 (20)	0.679
Education level, *n* (%) Primary school High school University	14 (40) 12 (34.3) 9 (25.7)	8 (20) 23 (57.5) 9(22.5)	0.092
Hand dominance, *n* (%) Right Left	32 (91.4) 3 (8.6)	34 (85) 6 (15)	0.393
MoCA score (Mean ± SD)	22.63 ± 3.97	28.86 ± 2.14	0.005 *
MoCA evaluation, *n* (%) Normal Low	25 (71.4) 10 (28.6)	38(95) 2 (5)	0.005 *
Follow-up period, months (Mean ± SD)	113 ± 15.5		
IgRT, *n* (%)	35 (100)		
Autoimmunity, *n* (%)	18 (51.4)		

* *p* ≤ 0.05 Abbreviations: BMI, body mass index; CVID, Common variable immunodeficiency; HC, healthy control; IgRT, immunoglobulin replacement therapy; MoCA, Montreal cognitive assessment; SD, standard deviation.

**Table 2:** Comparison of brain structures volume (cm^3^) and proportional (%) values between CVID and HC groups.

Brain Structures	CVID Group, (*n* = 36) Mean ± SD	HC Group, (*n* = 40) Mean ± SD	*p* Value
Tissue IC (cm^3^)	1409.45 ± 135.82	1416 ± 110.33	0.821
Tissue IC (%)	100 ± 0	100 ± 0	0.125
Tissue Brain (cm^3^)	1223.05 ± 210.61	1278.13 ± 117.06	0.009 *
Tissue Brain (%)	86.09 ± 3.97	88.77 ± 2.64	0.001 *
Tissue WM (cm^3^)	530.61 ± 76.79	524.94 ± 75.01	0.748
Tissue WM (%)	37.3 ± 3.08	35.91 ± 6.12	0.212
Tissue GM (cm^3^)	685.3 ± 81.39	717.51 ± 69.48	0.042 *
Tissue GM (%)	48.45 ± 3.08	50.76 ± 3.19	0.002 *
Cerebrum Total (cm^3^)	1055.99 ± 121.82	1096.88 ± 103.72	0.005 *
Cerebrum Total (%)	74.79 ± 3.76	77.18 ± 3.32	0.024 *
Cerebrum WM (cm^3^)	479.14 ± 63.64	461.15 ± 115.16	0.398
Cerebrum WM (%)	33.56 ± 3.01	33.52 ± 5.28	0.963
Cerebrum GM (cm^3^)	583.26 ± 70.34	657.48 ± 187.14	0.023 *
Cerebrum GM (%)	41.23 ± 2.64	45.71 ± 9.21	0.005 *
Cerebellum Total (cm^3^)	137.05 ± 17.86	142.58 ± 11.77	0.024 *
Cerebellum Total (%)	9.49 ± 0.67	9.95 ± 0.68	0.004 *
Cerebellum WM (cm^3^)	36.71 ± 7.61	32.94 ± 15.19	0.171
Cerebellum WM (%)	2.59 ± 0.46	2.33 ± 1.03	0.145
Cerebellum GM (cm^3^)	97.54 ± 12.87	107.54 ± 18.42	0.008 *
Cerebellum GM (%)	6.89 ± 0.66	7.6 ± 1.12	0.001 *
Hippocampus (cm^3^)	7.56 ± 0.93	8.63 ± 2.23	0.008 *
Hippocampus (%)	0.53 ± 0.05	0.59 ± 0.12	0.004 *
Amygdala (cm^3^)	1.19 ± 0.23	1.69 ± 0.57	0.000 *
Amygdala (%)	0.08 ± 0.01	0.12 ± 0.04	0.000 *
Thalamus (cm^3^)	10.38 ± 1.65	12.47 ± 3.27	0.001 *
Thalamus (%)	0.73 ± 0.08	0.88 ± 0.2	0.000 *
Caudate (cm^3^)	6.83 ± 1.15	8.89 ± 4.1	0.004 *
Caudate (%)	0.48 ± 0.06	0.62 ± 0.25	0.002 *
Putamen (cm^3^)	7.82 ± 1.3	9.78 ± 3.26	0.001 *
Putamen (%)	0.55 ± 0.06	0.69 ± 0.2	0.000 *
Globus Pallidus (cm^3^)	1.71 ± 0.4	1.98 ± 0.73	0.05 *
Globus Pallidus (%)	0.12 ± 0.02	0.14 ± 0.05	0.024 *
Accumbens (cm^3^)	0.6 ± 0.13	0.78 ± 0.27	0.001 *
Accumbens (%)	0.04 ± 0.01	0.05 ± 0.02	0.000 *
Brainstem (cm^3^)	22.8 ± 3.12	23.24 ± 2.63	0.513
Brainstem (%)	1.61 ± 0.14	1.65 ± 0.19	0.331
Tissue CSF (cm^3^)	199.25 ± 59.9	157.88 ± 53.53	0.003 *
Tissue CSF (%)	14.11 ± 4.08	11.13 ± 3.4	0.001 *
Lateral ventricles (cm^3^)	13.69 ± 8.88	9.01 ± 4.87	0.008 *
Lateral ventricles (%)	0.97 ± 0.65	0.64 ± 0.34	0.009 *

* *p* ≤ 0.05. Abbreviations: CSF, cerebrospinal fluid; CVID, Common variable immunodeficiency; GM, gray matter; HC, healthy control; IC, intracranial; SD, standard deviation; WM, white matter.

**Table 3:** Comparison of volumes (cm^3^) and proportional (%) values of right and left brain structures between CVID and HC groups.

	CVID (*n* = 35)			HC (*n* = 40)		
	Right Brain (Mean ± SD)	Left Brain (Mean ± SD)	*p* Value	Right Brain (Mean ± SD)	Left Brain (Mean ± SD)	*p* Value
Cerebrum total (cm^3^)	525.86 ± 74.72	528.45 ± 64.79	0.455	545.1 ± 65.52	546.78 ± 55.68	0.528
Cerebrum total (%)	37.48 ± 1.8	37.32 ± 2.03	0.243	38.67 ± 1.76	38.53 ± 1.63	0.094
Cerebrum GM (cm^3^)	292.67 ± 34.75	290.58 ± 35.73	0.010 *	329.13 ± 93.12	328.1 ± 94.29	0.097
Cerebrum GM (%)	20.69 ± 1.32	20.54 ± 1.34	0.017 *	23.19 ± 5.72	23.07 ± 5.56	0.035 *
Cerebrum WM (cm^3^)	237.78 ± 33.72	237.87 ± 35.8	0.935	233.41 ± 41.42	234.18 ± 51.51	0.814
Cerebrum WM (%)	16.78 ± 1.43	16.78 ± 1.61	0.971	16.9 ± 2.26	16.79 ± 2.64	0.146
Cerebellum total (cm^3^)	66.73 ± 8.03	67.47 ± 8.03	0.004 *	69.84 ± 7.38	70.37 ± 7.04	0.086
Cerebellum total (%)	4.72 ± 0.33	4.76 ± 0.33	0.023 *	4.94 ± 0.4	4.96 ± 0.37	0.343
Cerebellum GM (cm^3^)	47.9 ± 6.77	49.58 ± 6.32	0.000 *	53.36 ± 9.57	54.18 ± 8.94	0.012 *
Cerebellum GM (%)	3.39 ± 0.34	3.51 ± 0.33	0.000 *	3.77 ± 0.58	3.83 ± 0.55	0.009 *
CerebellumWM (cm^3^)	18.83 ± 3.77	17.89 ± 3.89	0.000 *	16.65 ± 7.67	16.29 ± 7.57	0.102
CerebellumWM (%)	1.33 ± 0.24	1.26 ± 0.23	0.000 *	1.17 ± 0.52	1.15 ± 0.52	0.109
Hippocampus (cm^3^)	3.85 ± 0.44	3.71 ± 0.53	0.008 *	4.32 ± 1.06	4.27 ± 0.97	0.458
Hippocampus (%)	0.27 ± 0.02	0.26 ± 0.03	0.011 *	0.3 ± 0.05	0.3 ± 0.05	0.001 *
Amygdala (cm^3^)	0.61 ± 0.12	0.58 ± 0.11	0.003 *	0.9 ± 0.36	0.64 ± 0.27	0.002 *
Amygdala (%)	0.04 ± 0.01	0.04 ± 0.01	0.07	0.06 ± 0.02	0.06 ± 0.02	0.000 *
Thalamus (cm^3^)	5.14 ± 0.69	5.26 ± 0.97	0.231	6.29 ± 1.73	6.15 ± 1.52	0.439
Thalamus (%)	0.36 ± 0.03	0.37 ± 0.06	0.588	0.44 ± 0.1	0.43 ± 0.09	0.273
Caudate (cm^3^)	3.44 ± 0.58	3.39 ± 0.58	0.030 *	4.47 ± 2.01	4.4 ± 2.03	0.152
Caudate (%)	0.24 ± 0.03	0.24 ± 0.03	0.143	0.31 ± 0.12	0.31 ± 0.12	0.069
Putamen (cm^3^)	3.94 ± 0.68	3.87 ± 0.65	0.079	4.93 ± 1.7	4.85 ± 1.56	0.052
Putamen (%)	0.28 ± 0.03	0.27 ± 0.03	0.109	0.35 ± 0.1	0.34 ± 0.09	0.040 *
Globus Pallidus (cm^3^)	0.87 ± 0.22	0.84 ± 0.2	0.12	0.98 ± 0.38	1 ± 0.37	0.193
Globus Pallidus (%)	0.06 ± 0.01	0.06 ± 0.01	0.038 *	0.07 ± 0.03	0.07 ± 0.03	0.236
Accumbens (cm^3^)	0.28 ± 0.06	0.32 ± 0.07	0.000 *	0.36 ± 0.13	0.4 ± 0.13	0.000 *
Accumbens (%)	0.02 ± 0	0.02 ± 0	0.183	0.03 ± 0.01	0.03 ± 0.01	0.001 *
Lateral ventricles (cm^3^)	5.85 ± 2.83	7.55 ± 5.46	0.009 *	4.37 ± 2.59	4.64 ± 2.5	0.258
Lateral ventricles (%)	0.43 ± 0.26	0.54 ± 0.42	0.021 *	0.31 ± 0.18	0.33 ± 0.18	0.264

* *p* ≤ 0.05. Abbreviations: CVID, Common variable immunodeficiency; GM, gray matter; HC, healthy control; SD, standard deviation; WM, white matter.

**Table 4:** Clinical variables and brain volume correlations.

Variable	Tissue Brain (cm^3^)	Cerebrum Total (cm^3^)	Tissue White Matter (cm^3^)	Tissue Gray Matter (cm^3^)	Tissue CSF (cm^3^)	Cerebellum Total (cm^3^)	Other
Diagnostic delay	NS	NS	NS	NS	NS	NS	NS
IgRT duration	NS	NS	NS	NS	r = 0.341 *p* = 0.041	NS	NS
Serum IgG level	NS	NS	NS	NS	NS	r = 0.351 *p* = 0.036	NS

Pearson correlation coefficients (r) are shown. Abbreviations: CSF, cerebrospinal fluid; IgRT, immunoglobulin replacement therapy; IgG, immunoglobulin G; NS: Not significant.

**Table 5:** Comparison of volumetric (cm^3^) and proportional (%) values of brain structures according to MoCA scores in the CVID group. (Mean ± SD).

	Normal MoCA Score (*n* = 25)	Low MoCA Score (*n* = 10)	*p* Value
Age	38.96 ± 10.65	37.20 ± 13.10	0.711
Tissue IC (cm^3^)	1442.85 ± 124.88	1325.95 ± 131.37	0.028 *
Tissue Brain (cm^3^)	1250.32 ± 125.48	1329.9 ± 347.2	0.496
Tissue Brain (%)	86.53 ± 3.84	85 ± 4.3	0.342
Tissue WM (cm^3^)	552.44 ± 66.76	476.06 ± 75.9	0.014 *
Tissue WM (%)	37.91 ± 2.83	35.78 ± 3.32	0.096
Tissue GM (cm^3^)	697.88 ± 77.37	653.84 ± 86.72	0.182
Tissue GM (%)	48.14 ± 3.18	49.22 ± 2.82	0.34
Cerebrum Total (cm^3^)	1084.91 ± 105.04	983.68 ± 136.02	0.054
Cerebrum Total (%)	75.12 ± 3.75	73.97 ± 3.85	0.433
Cerebrum WM (cm^3^)	495.73 ± 59.62	437.67 ± 56.07	0.014 *
Cerebrum WM (%)	34.2 ± 2.85	31.97 ± 2.93	0.057
Cerebrum GM (cm^3^)	593.27 ± 66.36	558.24 ± 77.3	0.228
Cerebrum GM (%)	40.92 ± 2.63	42 ± 2.62	0.287
Cerebellum Total (cm^3^)	138.08 ± 15.1	134.47 ± 24.22	0.669
Cerebellum Total (%)	9.53 ± 0.69	9.39 ± 0.62	0.565
Cerebellum WM (cm^3^)	38.07 ± 7.9	33.32 ± 5.86	0.064
Cerebellum WM (%)	2.63 ± 0.49	2.51 ± 0.39	0.481
Cerebellum GM (cm^3^)	100.09 ± 12.99	91.15 ± 10.6	0.048 *
Cerebellum GM (%)	6.9 ± 0.73	6.87 ± 0.44	0.891
Hippocampus Total (cm^3^)	7.74 ± 0.79	7.11 ± 1.13	0.137
Hippocampus Total (%)	0.53 ± 0.04	0.54 ± 0.07	0.934
Amygdala (cm^3^)	1.22 ± 0.22	1.1 ± 0.24	0.186
Amygdala (%)	0.08 ± 0.01	0.08 ± 0.02	0.882
Thalamus (cm^3^)	10.7 ± 1.36	9.56 ± 2.08	0.136
Thalamus (%)	0.74 ± 0.06	0.72 ± 0.11	0.577
Caudate (cm^3^)	6.84 ± 1.05	6.81 ± 1.44	0.961
Caudate (%)	0.47 ± 0.06	0.51 ± 0.07	0.122
Putamen (cm^3^)	7.99 ± 1.25	7.39 ±1.4	0.256
Putamen (%)	0.55 ± 0.06	0.56 ± 0.07	0.81
Globus Pallidus (cm^3^)	1.77 ± 0.41	1.57 ± 0.38	0.185
Globus Pallidus (%)	0.12 ± 0.02	0.12 ± 0.03	0.756
Accumbens (cm^3^)	0.61 ± 0.12	0.57 ± 0.14	0.426
Accumbens (%)	0.04 ± 0.01	0.04 ± 0.01	0.691
Brainstem (cm^3^)	23.22 ± 3.13	21.74 ± 3.01	0.212
Brainstem (%)	1.6 ± 0.13	1.64 ± 0.15	0.486
Tissue CSF (cm^3^)	200.53 ± 65.11	196.05 ± 47.26	0.823
Tissue CSF (%)	13.75 ± 4.03	15 ± 4.3	0.44
Lateral ventricles (cm^3^)	13.88 ± 8.99	13.23 ± 9.05	0.851
Lateral ventricles (%)	0.95 ± 0.59	1.03 ± 0.8	0.775

* *p* ≤ 0.05. Abbreviations: CSF, cerebrospinal fluid; CVID, Common variable immunodeficiency; GM, gray matter; IC, intracranial; MoCA, Montreal cognitive assessment; SD, standard deviation; WM, white matter.

**Table 6:** Comparison of the CVID Group According to the Presence of Autoimmune Conditions.

	Autoimmunity		
	Yes (*n* = 18)	No (*n* = 17)	*p* Value
Age (Mean ± SD)	37.5 ± 12.57	39.47 ± 9.91	0.609
Gender, *n* (%) Female Male	11 (61.1) 7 (38.9)	11 (64.7) 6 (35.3)	0.127
BMI (Mean ± SD)	25.54 ± 4.68	24.35 ± 3.90	0.416
BMI assessment, *n* (%) Normal Overweight Grade 1 obesity	10 (55.6) 4 (22.2) 4 (22.2)	11 (64.7) 4 (23.5) 2 (11)	0.702
Education level, *n* (%) Primary school High school University	6 (33.3) 7 (38.9) 5(27.8)	8 (47.1) 5 (29.4) 4 (23.5)	0.704
Hand dominance, *n* (%) Right hand Left hand	32 (91.4) 3 (8.6)	34 (85) 6 (15)	0.764
MoCA score (Mean ± SD)	22.44 ± 4.30	22.82 ± 3.71	0.782
MoCA assessment, *n* (%) Normal Low	7 (38.9) 11 (61.1)	3 (17.6) 14 (82.4)	0.164
Follow-up period, months (Mean ± SD)	140 ± 102	84.41 ± 71.41	0.072
Lymphocyte count, 10^3^/µL, (Mean ± SD)	1792.11 ± 93	1813.52 ± 56	0.826
Immunoglobulins, g/L IgA IgM IgG	0.73 ± 0.70 0.31 ± 0.28 3.88 ± 1.95	0.57 ± 0.60 0.50 ± 0.61 3.74 ± 2.1	0.472 0.258 0.841
Lymphocyte subgroups, % CD3^+^ T cells CD3^+^CD4^+^ T cells CD3^+^CD8^+^ T cells CD4^+^/CD8^+^ ratio CD19^+^ B cells CD19^+^27^+^IgD^−^ (SMB) CD16^+^56^+^ NK cells	79.22 ± 8.68 34.72 ± 10 41.94 ± 14.27 0.95 ± 0.50 8.45 ± 6.28 3.24 ± 2.7 6.96 ± 4.45	79.82 ± 9.7 38.58 ± 13.04 38.64 ± 15.58 1.23 ± 0.74 8.72 ± 6.52 5.00 ± 4.74 7.30 ± 4.67	0.849 0.335 0.519 0.211 0.900 0.192 0.831

Abbreviations: CVID, Common variable immunodeficiency; SD, standard deviation; BMI, body mass index; MoCA, Montreal cognitive assessment; Ig: immunoglobulin, CD, cluster of differentiation; SMB, switched memory B; NK, natural killer.

**Table 7:** Comparison of brain volumes (cm^3^) and proportional (%) values between CVID groups with and without autoimmunity and the HC group.

	CVID Group (*n* = 35)		HC Group (*n* = 40)		
	Autoimmunity Yes (a) (*n* = 17)	Autoimmunity No (b) (*n* = 18)	HC Group (c) (*n* = 40)	*p* Value	RG
Age	39.47 ± 9.91	37.5 ± 12.58	33.9 ± 9.41	0.147	
BMI	24.35 ± 3.91	25.55 ± 4.68	24.17 ± 3.63	0.464	
Tissue IC (cm^3^)	1394.93 ± 138.77	1423.17 ± 135.51	1416 ± 110.33	0.775	
Tissue Brain (cm^3^)	1266.45 ± 202.42	1279.29 ± 223.74	1278.13 ± 117.06	0.967	
Tissue Brain (%)	86.95 ± 3.83	85.28 ± 4.04	88.77 ± 2.64	0.001	
Tissue WM (cm^3^)	535.51 ± 82.2	525.99 ± 73.39	524.94 ± 75.01	0.887	
Tissue WM (%)	37.98 ± 3.24	36.65 ± 2.87	35.91 ± 6.12	0.357	
Tissue GM (cm^3^)	672.12 ± 68.14	697.74 ± 92.41	717.51 ± 69.48	0.116	
Tissue GM (%)	48.26 ± 2.79	48.63 ± 3.4	50.76 ± 3.19	0.009 *	ac
Cerebrum Total (cm^3^)	1050.12 ± 117.73	1061.53 ± 128.71	1096.88 ± 103.72	0.289	
Cerebrum Total (%)	75.27 ± 3.7	74.34 ± 3.86	77.18 ± 3.32	0.014 *	bc
Cerebrum WM (cm^3^)	487.36 ± 59.47	471.38 ± 68.12	461.15 ± 115.16	0.635	
Cerebrum WM (%)	34.33 ± 3.2	32.84 ± 2.71	33.52 ± 5.28	0.601	
Cerebrum GM (cm^3^)	570.07 ± 57.26	595.71 ± 80.45	657.48 ± 187.14	0.084	
Cerebrum GM (%)	40.94 ± 2.45	41.5 ± 2.85	45.71 ± 9.21	0.026 *	ac
Cerebellum Total (cm^3^)	140.7 ± 20.95	133.61 ± 14.1	142.58 ± 11.77	0.107	
Cerebellum Total (%)	9.65 ± 0.74	9.33 ± 0.57	9.95 ± 0.68	0.007 *	bc
Cerebellum WM (cm^3^)	37.26 ± 8.9	36.2 ± 6.37	32.94 ± 15.19	0.409	
Cerebellum WM (%)	2.66 ± 0.52	2.53 ± 0.41	2.33 ± 1.03	0.341	
Cerebellum GM (cm^3^)	97.56 ± 12.21	97.51 ± 13.81	107.54 ± 18.42	0.033 *	ac, bc
Cerebellum GM (%)	6.99 ± 0.53	6.8 ± 0.76	7.6 ± 1.12	0.007 *	bc
Hippocampus (cm^3^)	7.46 ± 1.11	7.65 ± 0.74	8.63 ± 2.23	0.035 *	ac, bc
Hippocampus (%)	0.53 ± 0.05	0.54 ± 0.04	0.59 ± 0.12	0.023 *	ac, bc
Amygdala (cm^3^)	1.14 ± 0.19	1.23 ± 0.25	1.69 ± 0.57	0.000 *	ac, bc
Amygdala (%)	0.08 ± 0.01	0.09 ± 001	0.12 ± 0.04	0.000 *	ac, bc
Thalamus (cm^3^)	10.06 ± 1.67	10.68 ± 1.63	12.47 ± 3.27	0.004 *	ac, bc
Thalamus (%)	0.72 ± 0.08	0.74 ± 0.07	0.88 ± 0.2	0.000 *	ac, bc
Caudate (cm^3^)	6.61 ± 0.99	7.04 ± 1.28	8.89 ± 4.1	0.019 *	bc
Caudate (%)	0.47 ± 0.06	0.49 ± 0.06	0.62 ± 0.25	0.011 *	ac, bc
Putamen (cm^3^)	7.75 ± 1	7.87 ± 1.57	9.78 ± 3.26	0.006 *	ac, bc
Putamen (%)	0.56 ± 0.05	0.55 ± 0.07	0.69 ± 0.2	0.001 *	ac, bc
Globus Pallidus (cm^3^)	1.66 ± 0.34	1.77 ± 0.46	1.98 ± 0.73	0.144	
Globus Pallidus (%)	0.12 ± 0.02	0.12 ± 0.03	0.14 ± 0.05	0.09	
Accumbens (cm^3^)	0.63 ± 0.12	0.58 ± 0.13	0.78 ± 0.27	0.003 *	ac, bc
Accumbens (%)	0.04 ± 0.01	0.04 ± 0.01	0.05 ± 0.02	0.001 *	ac, bc
Brainstem (cm^3^)	22.54 ± 3.65	23.03 ± 2.63	23.24 ± 2.63	0.709	
Brainstem (%)	1.61 ± 0.13	1.61 ± 0.14	1.65 ± 0.19	0.637	
Tissue CSF (cm^3^)	187.3 ± 58.14	210.54 ± 60.96	157.88 ± 53.53	0.005 *	bc
Tissue CSF (%)	13.46 ± 4.15	14.72 ± 4.04	11.13 ± 3.4	0.003 *	bc
Lateral ventricles (cm^3^)	12.37 ± 7.91	14.94 ± 9.76	9.01 ± 4.87	0.012 *	bc
Lateral ventricles (%)	0.9 ± 0.66	1.04 ± 0.64	0.64 ± 0.34	0.017 *	bc

* *p* ≤ 0.05. Abbreviations: BMI, body mass index; CSF, cerebrospinal fluid; CVID, Common variable immunodeficiency; GM, gray matter; HC, healthy control; IC, intracranial; RG: related groups; SD, standard deviation; WM, white matter.

## Data Availability

The data are available from the corresponding author upon easonable request.
